# Hyperspectral imaging for objective microcirculatory assessment in trauma patients

**DOI:** 10.1038/s41598-026-66067-2

**Published:** 2026-08-06

**Authors:** Marita Klein, Maik von der Forst, Nikolai Kaltschmidt, Frank Weilbacher, Louis de Re, Christine Leowardi, Hans Thomas Hölzer, Tim Niklas Bewersdorf, Maximilian Dietrich, Felix C. F. Schmitt, Markus A. Weigand, Erik Popp, Stephan Katzenschlager

**Affiliations:** 1 Medical Faculty Heidelberg, Department of Anesthesiology, Heidelberg University, Heidelberg, Germany; 2 Medical Faculty Heidelberg, Department of General, Visceral, and Transplantation Surgery, Heidelberg University, Heidelberg, Germany; 3 Medical Faculty Heidelberg, Division of Emergency Medicine, Heidelberg University, Heidelberg, Germany; 4 Medical Faculty Heidelberg, Heidelberg Trauma Research Group, Clinic for Trauma and Reconstructive Surgery, Center for Orthopedics, Trauma Surgery and Spinal Cord Injury, Heidelberg University, Heidelberg, Germany; 5 Department of Anesthesiology, Heidelberg University Hospital, Im Neuenheimer Feld 420, 69120 Heidelberg, Germany

**Keywords:** Hemodynamic instability, Microcirculation, Trauma, Resuscitation, Hyperspectral imaging, Biomarkers, Cardiology, Diseases, Medical research

## Abstract

Hyperspectral imaging (HSI) is a non-invasive technology capable of assessing microcirculatory impairment. This study evaluated whether HSI can detect early microcirculatory alterations in trauma patients and differentiate between those with hemodynamic instability or stability during initial trauma resuscitation. In this prospective single-center observational study at a German level I trauma center (01/2023–10/2024), patients underwent HSI (TIVITA^®^ 2.0) at three time points: during the primary survey, after resuscitation room care, and 24 h after admission. HSI approximates four microcirculatory parameters: tissue oxygenation saturation (StO₂), tissue water index (TWI), tissue hemoglobin index (THI), and near-infrared perfusion index (NIR). Hemodynamic instability was defined as systolic blood pressure < 90 mmHg for > 10 min, catecholamine use > 10 min, or pronounced clinical signs of hypoperfusion. The primary outcome was the difference in StO₂ between patients with and without hemodynamic instability at the initial HSI measurement. Secondary outcomes included comparison of all HSI parameters between groups, correlations with Injury Severity Score (ISS), biomarkers (lactate, bicarbonate, base excess), and mortality. Statistical analyses comprised t-tests, receiver operator curve (ROC) analyses, diagnostic accuracy, and DeLong’s test. A total of 163 patients were analyzed (18 hemodynamic instability, 145 hemodynamic stability). No significant difference in initial StO₂ was observed between groups (hemodynamic instability: 56.50 (SD 17.08) vs. hemodynamic stability: 59.48 (SD 10.92); *p* = 0.311). Likewise, TWI, THI, and NIR showed no significant differences. Initial elevated lactate levels (≥ 2 mmol/L) were associated with significantly lower StO_2_ levels (*p* = 0.002). ROC analysis of StO₂ for predicting in-hospital mortality showed an area under the curve of 0.677 (95% CI 0.529–0.825). The optimal mortality cut-off (StO₂ ≤ 54%) demonstrated a sensitivity of 0.70, specificity of 0.66, positive predictive value of 0.22, and negative predictive value (NPV) of 0.94. AUCs of StO₂, lactate, bicarbonate, and base excess overlapped, with no significant pairwise differences. HSI did not discriminate between hemodynamically unstable and stable patients at initial trauma assessment but correlated well with high lactate levels. However, its high NPV for mortality suggests potential value as an objective, non-invasive tool for early trauma team downgrading.

*Trial registration: *German Clinical Trials Register (DRKS ID DRKS00030986) on 27th December 2022.

**Supplementary Information:**

The online version contains supplementary material available at 10.1038/s41598-026-66067-2.

## Introduction

Trauma remains one of the leading causes of death worldwide. Despite improvements over the last decades, hemorrhage and exsanguination account for a large proportion of these deaths^1–4^. Acute hemorrhage following major trauma induces hemorrhagic shock, characterized by systemic hypoperfusion and tissue hypoxia, potentially progressing to multi-organ failure and death without timely intervention^4^. In the early stages of shock, microcirculatory impairment is present^5,6^. At the same time, macrocirculatory parameters, such as systolic blood pressure, may persist within physiological ranges due to vital compensatory mechanisms^7,8^. Standard monitoring primarily captures indicators of macrocirculatory function. However, these standard vital parameters are limited in their ability to reliably detect early hemodynamic instability^8–10^. As a result, impaired tissue oxygenation might remain undetected until cardiovascular decompensation becomes clinically apparent. Therefore, assessing microcirculatory function in severely injured patients can support the early identification of those at risk, enabling timely interventions and improving patient outcomes^8,9,11^. Despite its clinical relevance, there is currently no standardized objective method for assessing microcirculatory function. Evaluation of the capillary refill time (CRT) is a well-established clinical approach for rapid assessment of centralization of circulation in critically ill patients and in trauma care. However, CRT shows considerable interrater variability and is influenced by patient and environmental factors, such as age, sex, skin color, as well as ambient and body temperature^12–14^. Although commonly used in clinical practice, elevated plasma lactate levels remain a non-specific surrogate of anaerobic metabolism, offering only limited insight into tissue perfusion and oxygenation^14^. Hyperspectral imaging (HSI) is a non-invasive technique that quantifies tissue oxygenation and perfusion, as well as tissue hemoglobin and water content. Previous studies demonstrated that HSI identifies tissue oxygenation and perfusion deficits after blood loss and monitors the microcirculatory response to both fluid and vasopressor resuscitation in a porcine model of hemorrhagic shock^15,16^. In septic patients, HSI showed promising clinical value by revealing a characteristic microcirculatory pattern associated with disease severity and mortality^17^. A feasibility study demonstrated the integration of HSI into trauma resuscitation room workflows during the primary survey^18^. Despite promising results in other clinical fields, the role of HSI in emergency medicine, particularly in trauma, remains poorly investigated. Therefore, this study aims to examine HSI parameters in trauma patients admitted to the resuscitation room of a level I trauma center in Germany. The objective of this study is to compare HSI-derived microcirculatory parameters between trauma patients with and without hemodynamic instability. We hypothesize that differences in tissue oxygenation will be detectable between these groups.

## Methods

### Study design and setting

This prospective, single-center observational study was conducted in the resuscitation room of a level I trauma center in Germany, which treats approximately 500 trauma patients annually. The standard trauma team is organized in an interdisciplinary and multiprofessional structure, including, at a minimum, one anesthesiologist, one anesthesia nurse, one trauma surgeon, one surgical nurse, one visceral surgeon, one radiologist, and one radiology technician. Depending on the pattern of injury, additional specialties, such as neurosurgery, vascular surgery, or pediatric surgery, may be involved. Trauma team activation followed the criteria of the German S3 guideline on polytrauma management, including a lower threshold for geriatric patients (Supplement Table 1)^4^. The study protocol was reviewed and approved by the Ethics Committee of the Medical Faculty of Heidelberg University, Germany (ID: S-671/2022). Before patient enrolment, the study was registered at the German Clinical Trial Register (www.drks.de – DRKS-ID: DRKS00030986) on 27th December 2022. This investigation was financed by institutional resources of the University Hospital Heidelberg, Clinic for Anesthesiology. All methods were carried out in accordance with relevant guidelines and regulations. The reporting of this study followed the Strengthening the Reporting of Observational Studies in Epidemiology (STROBE) Statement^19^ (Supplement Checklist 1).

Inclusion criteria:

All trauma patients admitted to the resuscitation room following the criteria of the German S3 guideline on polytrauma management were included, regardless of age or mechanism of trauma. Known prisoner status or missing consent by the patient or a legal representative resulted in exclusion from the study.

### Enrolment and consent

Patients were initially enrolled in the trial under an emergency exemption, which was approved by a member of the study team and an independent physician. Written informed consent was obtained from conscious patients after the initial treatment. In unconscious patients, a legal representative was installed and asked for informed consent as soon as possible. As soon as the patient regained consciousness, informed consent was obtained. Patients who died during the initial treatment were enrolled without further consent. According to local regulations, children ≥ 7 years of age had to provide their assent, in addition to the written informed consent from their caregivers. For children aged < 7 years, only the caregiver had to provide written informed consent.

### Study intervention

Following handover of the patient from the emergency medical service (EMS) to the in-hospital team, a primary assessment was initiated in accordance with established trauma protocols. In total, three HSI measurements were obtained at different stages of in-hospital patient care. The first HSI measurement was performed during the primary survey in the trauma resuscitation room. Immediately after the resuscitation room care was completed, the second HSI measurement was performed, either before or immediately after, the patient’s transfer to the operating room (OR), intensive care unit (ICU), or ward. When the patient was still in the hospital, a third HSI image was taken 24 h after the initial admission to the resuscitation room. During and after the resuscitation room phase, demographic and clinical data, relevant interventions, and medication were documented. A comprehensive overview of all collected data points is provided in the Supplements (Supplement Table 2).

### Group classification

The study cohort was divided into two groups based on clinical presentation prior to each HSI measurement, resulting in possible crossovers between timepoints and groups. This resulted in dynamic allocation based on the patient’s physiological condition at the respective timepoint. Those with hemodynamic instability and those with hemodynamic stability. Assignment to the hemodynamic instability group was based on the following clinical criteria, which were defined by the study team before patient enrolment:

1. Systolic blood pressure < 90 mmHg for > 10 min, or.
2. Continuous or repeated administration of catecholamines for a period of > 10 min, or.
3. Pronounced signs of skin hypoperfusion that are characterized by coldness, pallor, and CRT > 2 s.

### Hyperspectral Imaging

As in the pilot trial for this study, all HSI measurements were taken using the halogen-based camera Tivita^®^ Tissue System (Diaspective Vision GmbH, Am Salzhaff, Germany)^18^. The device captures reflected light across the visible and near-infrared (NIR) spectral ranges, specifically from 500 to 1000 nm, at 5 nm intervals for each pixel, resulting in 100 spectral data points per pixel at a resolution of 640 × 480 pixels. The visible spectrum is used for the generation of standardized Red-Green-Blue (RGB) images and the analysis of superficial tissue components (penetration depth ≈ 1 mm). In contrast, the NIR spectral range allows assessment of deeper tissue layers (penetration depth ≈ 4–6 mm). The system produces a three-dimensional hyperspectral dataset, containing both spatial and spectral dimensions, which enables detailed characterization of physiological and biochemical tissue properties. Based on this dataset, the following HSI parameters are calculated using their respective spectral wavelengths:

- Tissue oxygenation saturation (StO_2_) wavelength range: 500–650 and 700–815 nm, indicated in percent (0–100%), penetration depth of ≈ 1 mm.
- Near infrared perfusion index (NIR) wavelength range: 655–735 and 825–925 nm, indicated in predefined arbitrary units (0–100), penetration depth of ≈ 4–6 mm.
- Tissue hemoglobin index (THI) wavelength range: 530–590 and 785–825 nm, indicated in predefined arbitrary units (0–100).
- Tissue water index (TWI) wavelength range: 880–900 and 955–980 nm indicated in predefined arbitrary units (0–100).

These four HSI parameters are then visualized as color-coded images, with red/yellow tones indicating higher values (50–100) and green/blue tones representing lower values (0–50). To enable quantitative analysis, the integrated software allows the definition of regions of interest (ROIs), from which mean values are calculated. Currently, no clinical cutoff points for impaired microcirculation have been established. A circular ROI was defined for each measurement site (routine ROI = 45 units, only deviating, if necessary, due to hand size, superficial skin defects, visible contamination, external medical devices, or dressings). The palm of the hand served as the standard site for HSI measurements; when this site was inaccessible due to bilateral upper extremity trauma, an alternative site was selected on the medial thigh, above the patella. The system was used in accordance with the manufacturer’s operating instructions. Thresholds were predefined by the manufacturer’s software. Additionally, device calibrations were performed daily to ensure reliable measurements and improve comparability under varying conditions. To account for potential optical confounding by skin pigmentation, the Fitzpatrick skin phototype was documented, as epidermal melanin can alter light absorption and backscattering characteristics relevant to hyperspectral imaging and has been shown to influence HSI-derived tissue indices, including StO₂ and THI^20^.

### HSI measurements

One study team member selected the appropriate measurement site. Before each measurement, the chosen site was cleaned of visible blood and major contaminants. Local surface temperature and CRT were assessed in the same area where the HSI measurement was performed. The camera system was positioned approximately 50 cm above the measurement site, using the integrated optical distance guide, and the integrated stray-light indicator was monitored (Supplement Fig. 1). The measurement site was shielded from direct exposure to strong light sources, and no further modifications to ambient lighting were made. HSI image acquisition, data processing, and rendering were completed within approximately 15 s. Additionally, quantitative analysis was conducted within seconds by defining an ROI, from which numerical values were automatically calculated (Supplement Fig. 3). The entire HSI measurement process, including site preparation, camera positioning, and analysis, was completed in approximately 1 min, enabling rapid, non-invasive bedside evaluation of microcirculatory status. According to the study protocol, HSI measurements were primarily intended to be obtained from the palm of the hand. Measurements from the medial thigh were predefined as an acceptable alternative and did not lead to exclusion. However, post hoc analysis revealed systematically lower HSI values in the medial thigh than in the palmar hand. Consequently, the small number of thigh measurements was excluded from the analysis to improve comparability and reduce bias.

### Primary and secondary outcomes

The primary objective of this study was to detect a difference in StO_2_ between patients with and without hemodynamic instability using HSI in trauma patients. Secondary outcomes included comparisons of all four HSI parameters between patients with and without hemodynamic instability, diagnostic accuracy of HSI parameters for mortality, and comparison to various biomarkers, such as lactate, bicarbonate, and base excess (BE).

### Sample size

Based on previous experience, approximately 300 patients could be screened over the planned 12-month recruitment period, with about 85% being eligible for the study. A group distribution of approximately 1:5 (hemodynamic instability vs. stability) was anticipated. This corresponds to an expected sample size of 50 patients in the hemodynamic instability group and 200 patients in the hemodynamic stability group. According to data from the preliminary study^18^, the primary endpoint of StO_2_ follows a standard normal distribution, with a standard deviation of about 10. Based on the assumed total sample size of 250 patients, the estimated width of the two-sided 95% confidence interval for the difference in mean StO_2_ values between the two groups is approximately 6.82 units.

### Data collection and documentation

Data from the EMS, trauma resuscitation room, and in-hospital treatment were collected from digital or paper-based records. All data were pseudonymized and entered into an electronic database (Microsoft Excel^®^, Microsoft 365, Version 2511; Microsoft Corporation, Redmond, WA, USA). All injuries were coded according to the Abbreviated Injury Scale (AIS), and injury severity was assessed by calculating the Injury Severity Score (ISS), which ranges from 1 to 75 points^21^. Revised Trauma Score was calculated using the formula: GCS * 0.9368 + Systolic Blood Pressure * 0.7326 + Respiratory Rate * 0.2908. The shock index was calculated as Heart Rate/Systolic Blood Pressure.

### Statistical analysis

All analyses were based on the respective study group allocation at different timepoints, since the allocation was not randomized but determined by predefined clinical criteria. Demographic and baseline clinical characteristics were presented as means with standard deviations (SD) for parametric data or medians with 25th and 75th percentiles (interquartile range, IQR) for non-parametric continuous data, and as counts and percentages for categorical variables. For the primary endpoint of StO_2_ differences between the two groups, normality was assessed visually using histograms; consequently, a Student’s t-test with 95% confidence intervals (CI) was used. Categorical parameters were analyzed using the chi-square test or Fisher’s exact test. Bonferroni and Benjamini–Hochberg corrections were applied in the case of multiple testing. In septic patients, a lactate level > 2 mmol/l is used as a cut-off to identify critically ill patients. Therefore, we performed an exploratory analysis of HSI parameters in patients, irrespective of the assigned group, stratified by lactate levels (≤ 2 vs. > 2 mmol/l).

Longitudinal changes in HSI-derived StO₂ were analyzed using a linear mixed-effects model with time point as a categorical fixed effect and a subject-specific random intercept to account for repeated measurements within individuals. The model was estimated using restricted maximum likelihood and included all available observations, thereby accommodating incomplete follow-up under the missing-at-random assumption. Model-based estimated marginal means with 95% CI were calculated for each time point, and pairwise comparisons were adjusted for multiple testing using the Bonferroni method.

The diagnostic accuracy of HSI parameters for hemodynamic instability and mortality was assessed by calculating sensitivity, specificity, positive predictive value (PPV), and negative predictive value (NPV). Because higher StO₂ values reflect better physiological status, lower values were considered test positive. Discrimination was assessed using receiver-operating characteristic (ROC) analysis with calculation of the area under the curve (AUC) and corresponding 95% CI. The optimal cut-off value was determined by maximizing the Youden index (sensitivity + specificity – 1). At the calculated threshold (StO₂ ≤ 54%), we report the sensitivity, specificity, PPV, and NPV. Proportion CIs were computed using Wilson’s method. ROC curves were also constructed for lactate, bicarbonate, and base excess, and the AUCs with 95% CIs were calculated. Pairwise comparisons of AUCs were performed using DeLong’s test for two correlated ROC curves, as implemented in the roc.test function of the pROC package (version 1.19.0.1) in R (version 4.3.1, Vienna, Austria). A two-sided significance level of 5% was considered statistically significant. Clinical utility was evaluated using decision-curve analysis according to Vickers and Elkin. A logistic regression model using initial StO₂ as a continuous predictor generated individual probabilities of in-hospital mortality, and net benefit was calculated across threshold probabilities of 1–20% and compared with strategies of retaining full trauma-team resources for all or no patients. Model optimism was assessed using 1,000 bootstrap repetitions, with the model refitted in each sample and the average difference between bootstrap-sample and test performance used to obtain optimism-corrected net benefit and pointwise 95% confidence intervals. All other statistical analyses were conducted using IBM SPSS Statistics version 30.0 (IBM Corp., Armonk, NY, USA), along with Python plug-in extensions where appropriate.

## Results

Due to the unanticipated slow recruitment process, the trial had to be stopped early.

### Demographics

**Fig. 1:**
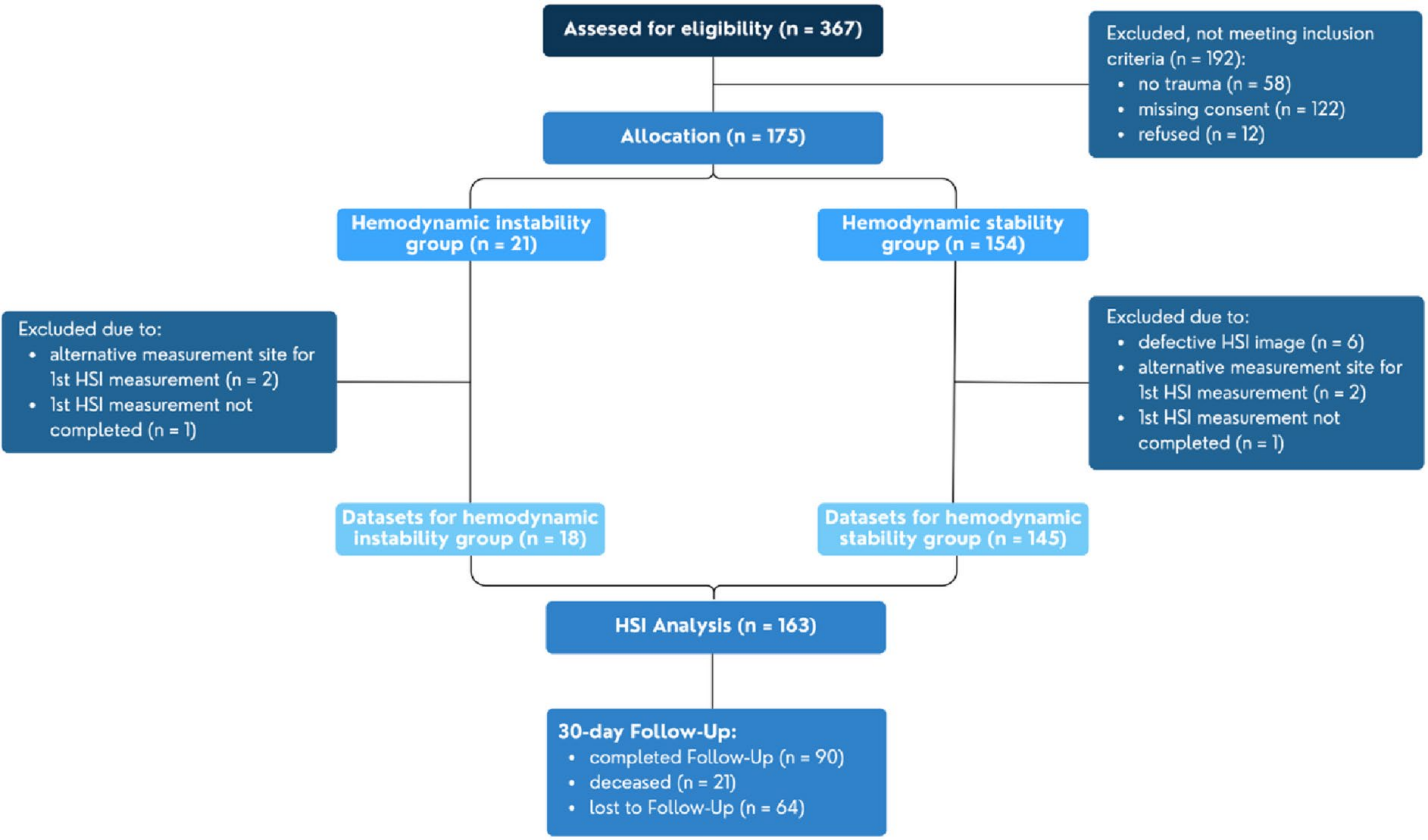
Study flow diagram. *HSI* Hyperspectral Imaging.

A total of 367 trauma patients were screened for eligibility between January 1 st, 2023, and October 11th, 2024. Group allocation was conducted for 175 patients. Repeated assessment of hemodynamic instability criteria at each measurement time point led to partial patient crossover between groups as their hemodynamic status evolved. Overall, 12 patients had to be excluded after group allocation, with six measurements excluded because an alternative measuring site on the medial thigh was used, which is not comparable to the standard site. Six cases had to be excluded due to a defective HSI image. The 30-day follow-ups were completed for 111 patients. This resulted in 163 datasets being included in the final analysis (Fig. 1).

Baseline patient demographics, underlying trauma mechanisms, vital signs, blood gas parameters, and outcomes for both groups are summarized in Tables 1 and 2. In the hemodynamic instability group, the mean age was 61 years (SD 26), compared to 51 years (SD 22) in the hemodynamic stability group. Approximately 50% in the hemodynamic instability group and 30% of the patients in the hemodynamic stability group were female. The mean ISS differed significantly between the groups, with 30 (SD 18) in the hemodynamic instability group and 11 (SD 10) in the hemodynamic stability group (*p* < 0.001). Likewise, the Revised Trauma Score was substantially lower in patients presenting with hemodynamic instability (Table 1). In the hemodynamic instability group, the most frequent mechanisms of trauma were pedestrian accidents, isolated traumatic brain injuries, falls from heights ≥ 3 m, domestic ground-level falls, and penetrating trauma. In the hemodynamic stability group, bicycle, motor vehicle, and pedestrian accidents, as well as stair falls, were the most prevalent. The mean Fitzpatrick scale was not different between groups, although more patients had a Fitzpatrick scale of 3–4 in the hemodynamic stability group.

On admission, most vital signs and blood gas parameters differed significantly between the two groups. In addition, patients in the hemodynamic instability group had a significantly higher in-hospital mortality (Table 2).

**Table 1:** Baseline demographics and patient characteristics.

	Hemodynamic instability (*n* = 18)	Hemodynamic stability (*n* = 145)	Overall (*n* = 163)
Age, mean (SD)	61 (26)	51 (22)	52 (23)
Female, n (%)	9 (50)	44 (30)	53 (32)
ISS, mean (SD) *	30 (18)	11 (10)	13 (13)
RTS, mean (SD) *	4.11 (1.32)	7.47 (1.11)	7.09 (1.56)
Blunt mechanism, n (%)			
Motor vehicle accident	1 (5.5)	34 (23.4)	35 (21.5)
Motorcycle accident	0	12 (8.2)	12 (7.3)
Bicycle accident	1 (5.5)	25 (17.1)	26 (15.9)
Pedestrian accident**	4 (22)	3 (2.1)	7 (4.3)
Fall ≥ 3 m	2 (11)	17 (11.7)	19 (11.6)
Fall < 3 m	0	9 (6.2)	9 (5.5)
Stair fall	1 (5.5)	13 (8.9)	14 (8.5)
Domestic ground-level fall	2 (11)	10 (6.8)	12 (7.3)
Isolated TBI	3 (16.6)	5 (3.4)	8 (4.9)
Other	2 (11)	13 (8.9)	15 (9.1)
Penetrating trauma, n (%)			
Stab wound	2 (11)	4 (2.7)	6 (3.7)
Gunshot wound	0	1 (0.7)	1 (0.6)
Prehospital Medication, n (%)			
Noradrenaline *	15 (83.3)	1 (0.7)	16 (9.8)
Adrenaline *	7 (38.9)	0	7 (4.3)
Other catecholamines	0	4 (2.8)	4 2.5)
Sufentanil	6 (33.3)	44 (30.3)	50 (30.7)
Fentanyl	1 (5.6)	16 (11)	17 10.4)
Piritramid	1 (5.6)	13 (9)	14 (8.6)
Midazolam **	10 (55.6)	34 (23.4)	44 (27)
Propofol	3 (16.7)	4 (2.8)	7 (4.3)
Esketamine	5 (27.8)	31 (21.4)	36 (22.1)
Rocuronium *	9 (50)	10 (6.9)	19 (11.7)
Other sedatives or analgesics	0	12 (8.3)	12 (7.4)
Other medication	11 (61.1)	59 (40.7)	70 43)
Crystalloids [ml], mean (SD)	1744 (1967)	466 (334)	
Colloids **	3 (16.7)	1 (0.7)	4 (2.5)
Red blood cell transfusion *	8 (44.4)	1 (0.7)	9 (5.5)
Other hemostatic medication *	5 (27.8)	0	5 (3.1)
Therapeutic interventions			
Needle decompression (prehospital)	1 (5.6)	0	1 (0.6)
Needle decompression (resuscitation room)	0	0	0
Pelvic sling (prehospital)	6 (33.3)	20 (13.8)	26 (16)
Pelvic sling (resuscitation room)	1 (5.6)	1 (0.7)	2 (1.2)
Tourniquet (prehospital)	2 (11.1)	1 (0.7)	3 (1.8)
Tourniquet (resuscitation room)	1 (5.6)	0	1 (0.6)
Thoracostomy (prehospital) *	6 (33.3)	1 (0.7)	7 (4.3)
Thoracostomy (resuscitation room) **	2 (11.1)	0	2 (1.2)
REBOA (prehospital)	0	0	0
REBOA (resuscitation room)	1 (5.6)	0	1 (0.6)
Resuscitative thoracotomy (prehospital)	1 (5.6)	0	1 (0.6)
Resuscitative thoracotomy (resuscitation room)	1 (5.6)	0	1 (0.6)
Pericardiocentisis (prehospital)	1 (5.6)	0	1 (0.6)
Pericardiocentisis (resuscitation room)	0	0	0
Endotracheal Intubation (prehospital) *	12 (66.7)	12 (8.3)	24 (14.7)
Endotracheal Intubation (resuscitation room)	2 (11.1)	2 (1.4)	4 (2.5)
Other (prehospital) *	8 (44.4)	3 (2.1)	11 (6.7)
Other (resuscitation room) **	15 (83.3)	75 (51.7)	90 (55.2)

*SD* standard deviation, *ISS* injury severity score, *RTS* Revised Trauma Score, *TBI* traumatic brain injury, *REBOA* Resuscitative Endovascular Balloon Occlusion of the Aorta. Significance was determined using Welch’s two-sample t-test for mean (SD) and two-sided Fisher’s exact test for categorical variables. Bonferroni and Benjamini–Hochberg corrections for multiple testing were applied: *p-value < 0.001; ** p-value < 0.05.

**Table 2:** Vital parameters, blood gas analysis on admission, and outcome.

	Hemodynamic instability (n = 18)	Hemodynamic stability (n = 145)	p-value
Vital parameters on admission, mean (SD)			
Respiratory rate [1/min]*	17.7 (4.5)	17.9 (4.8)	0.84
SpO_2_ [%]	93 (13)	97 (3)	0.001
Heart rate [1/min]	94 (26)	91 (18)	0.54
CRT [sec]**^,+^	2.6 (1.6)	1.7 (0.9)	< 0.001
Systolic blood pressure [mmHg]	92 (28)	137 (25)	< 0.001
GCS, mean (SD)	4.4 (3)	13.6 (3.4)	< 0.001
GCS = 3, n (%)	14 (78)	12 (8.3)	-
GCS ≤ 8, n (%)	15 (83)	14 (9.7)	-
GCS ≥ 14, n (%)	0 (0)	122 (84.1)	-
Palm skin temperature [°C]^§,+^	29 (4)	30 (3)	0.47
Body temperature [°C]***^,++^	35.8 (1.1)	36.6 (0.8)	0.004
Fitzpatrick Scale [1-6], mean (SD)*	2.2 (0.8)	1.3 (0.7)	0.452
Fitzpatrick score 1-2, n (%)	16 (89)	108 (76.1)	-
Fitzpatrick score 3-4, n (%)	1 (5.5)	33 (23.2)	-
Fitzpatrick score 5-6, n (%)	1 (5.5)	1 (0.7)	-
Blood gas parameters, mean (SD)			
pH**^,+^	7.21 (0.17)	7.37 (0.07)	< 0.001
Base excess [mmol/l]^§,+^	-9 (7)	-2 (3)	< 0.001
Lactate [mmol/l]^§§^	5.66 (3.89)	2.44 (1.67)	< 0.001
Hb [g/dl]**	10.7 (2.1)	13.2 (1.8)	< 0.001
Outcome, n (%)			
OR	15 (83.3)	49 (33.8)	< 0.001
ICU	17 (94.4)	64 (44.1)	< 0.001
In-hospital Death	10 (55.6)	7 (4.8)	< 0.001

### Hyperspectral imaging

**Fig. 2:**
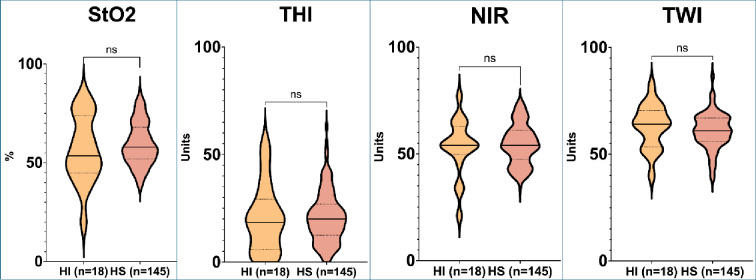
Violin Plot comparing the HSI parameters between the hemodynamic stability and instability groups at the initial measurement. *HI* Hemodynamic instability, *HS* Hemodynamic stability, *StO*_*2*_ tissue oxygenation saturation, *THI* tissue hemoglobin index, *NIR* near infrared perfusion index, *TWI* tissue water index, *ns* no statistical significance.

No significant difference in StO₂ values was observed between the two study groups during the first HSI assessment in the trauma resuscitation room. Similarly, none of the other HSI-derived parameters showed a significant difference between the two groups at the initial measurement (Table 3; Fig. 2).

**Table 3:** Primary Outcome of StO_2_ between the two groups.

	Hemodynamic instability (*n* = 18)	Hemodynamic stability (*n* = 145)	Difference (95% CI)	*p*-value
StO₂ [%], mean (SD)	56.50 (17.08)	59.48 (10.92)	3.00 (−2.81 to 8.76)	0.311
THI [U], mean (SD)	20.28 (15.80)	20.81 (11.86)	0.53 (−5.56 to 6.61)	0.864
NIR [U], mean (SD)	53.17 (13.21)	54.42 (9.36)	1.25 (−3.60 to 6.11)	0.611
TWI [U], mean (SD)	62.94 (10.34)	60.93 (8.47)	−2.01 (−6.30 to 2.27)	0.355

*StO*_*2*_ tissue oxygenation saturation, *THI* tissue hemoglobin index, *NIR* near infrared perfusion index, *TWI* tissue water index, *SD* standard deviation, *CI* confidence interval.

Analysis of the second and third HSI measurement time points (Supplement Tables 3 and 4) showed a significant difference in StO₂ at time point three between the hemodynamic instability and stability groups.

To assess differences in HSI measurements among patients with microcirculatory alterations, patients were divided into two groups based on their initial lactate value: > 2 mmol/L and ≤ 2 mmol/L. Higher lactate levels were associated with significantly lower StO_2_ values (Table 4). Further, differences in HSI measurements by shock index were evaluated, with no differences (Supplement Table 5).

**Table 4:** Comparison of HSI values according to Lactate values.

	Lactate ≤ 2 mmol/l (*n* = 75)	Lactate > 2 mmol/l (*n* = 79)	Difference (95% CI)	*p*-value
StO₂ [%], mean (SD)	61.64 (10.91)	55.90 (11.28)	5.74 (2.20 to 9.28)	0.002
THI [U], mean (SD)	20.36 (12.10)	21.10 (12.90)	−0.74 (−4.73 to 3.24)	0.714
NIR [U], mean (SD)	54.65 (9.96)	53.01 (9.45)	1.64 (−1.45 to 4.73)	0.296
TWI [U], mean (SD)	60.43 (9.35)	61.96 (8.06)	−1.53 (−4.31 to 1.24)	0.276

*StO*_*2*_ tissue oxygenation saturation, *THI* tissue hemoglobin index, *NIR* near infrared perfusion index, *TWI* tissue water index, *SD* standard deviation, *CI* confidence interval.

In a linear mixed-effects model with time as a categorical fixed effect and a subject-specific random intercept, HSI-derived StO₂ did not differ significantly across time points (F(2, 228) = 2.26, *p* = 0.107). Estimated marginal means were 59.16 (95% CI 57.44 to 60.88) at the first HSI measurement, 57.46 (95% CI 55.73 to 59.19) at the second HSI measurement, and 59.73 (95% CI 56.83 to 62.63) at the third HSI measurement, with no significant pairwise differences after Bonferroni adjustment.

### Diagnostic accuracy

**Fig. 3:**
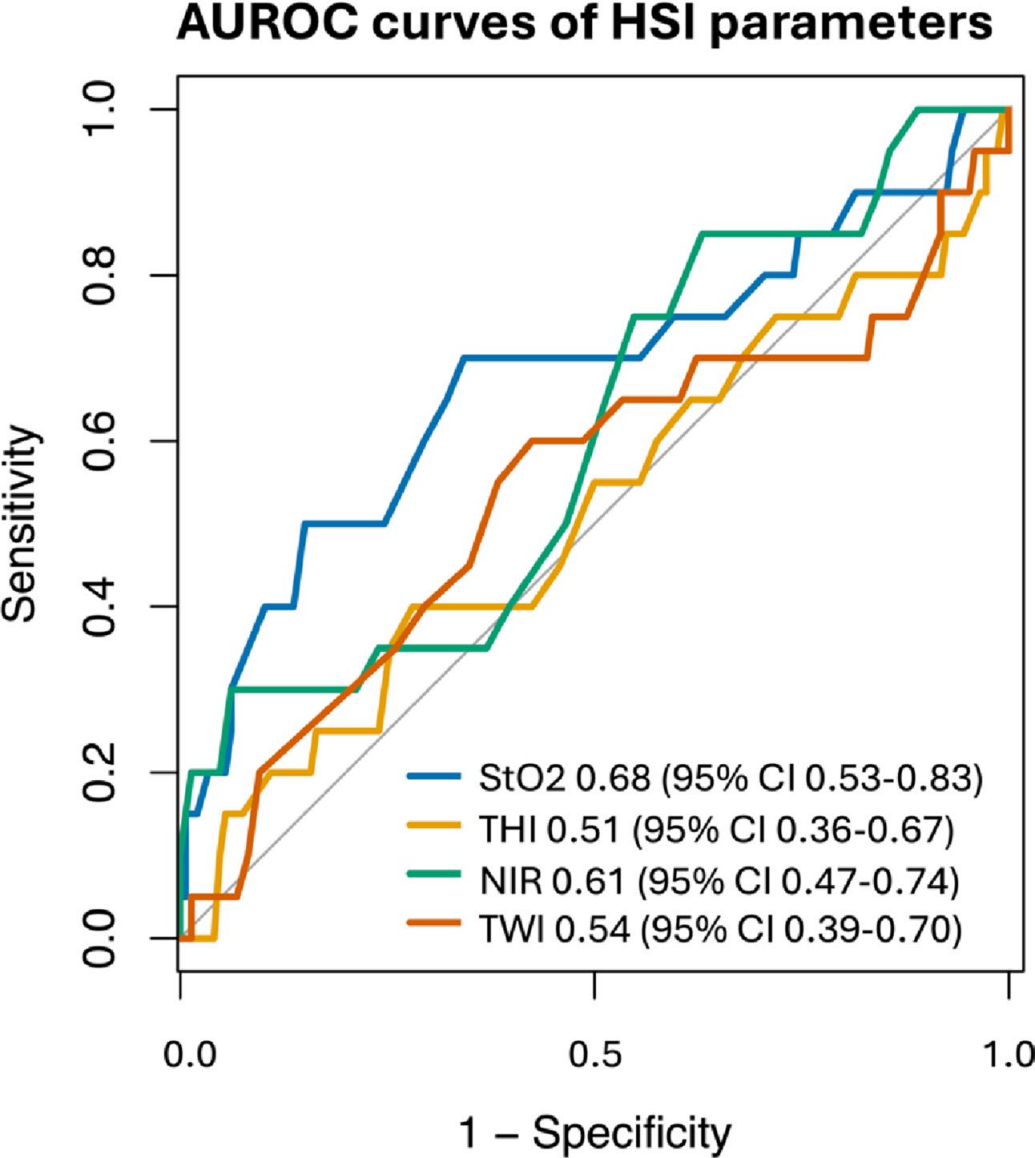
AUROC curves of HSI parameters for the prediction of in-hospital mortality. *AUROC* area under the receiver operating curve, *HIS* Hyperspectral Imaging, *StO*_*2*_ tissue oxygenation saturation, *THI* tissue hemoglobin index, *NIR* near infrared perfusion index, *TWI* tissue water index, *CI* confidence interval.

ROC analysis of initial HSI-derived StO₂ for prediction of in-hospital mortality yielded an AUC of 0.677 (95% CI 0.529–0.825). The AUC for THI was 0.514 (95% CI 0.362–0.665), for NIR was 0.605 (95% CI 0.468–0.742), and for TWI was 0.542 (95% CI 0.386–0.697), respectively (Fig. 3). The optimal mortality threshold for StO₂ (≤ 54%) showed a sensitivity of 0.70, a specificity of 0.66, a PPV of 0.22, a NPV of 0.94, a positive likelihood ratio of 1.92 (95% CI 1.26–2.91), and a negative likelihood ratio of 0.53 (95% CI 0.28–1.03) (Supplement Table 6).

**Fig. 4:**
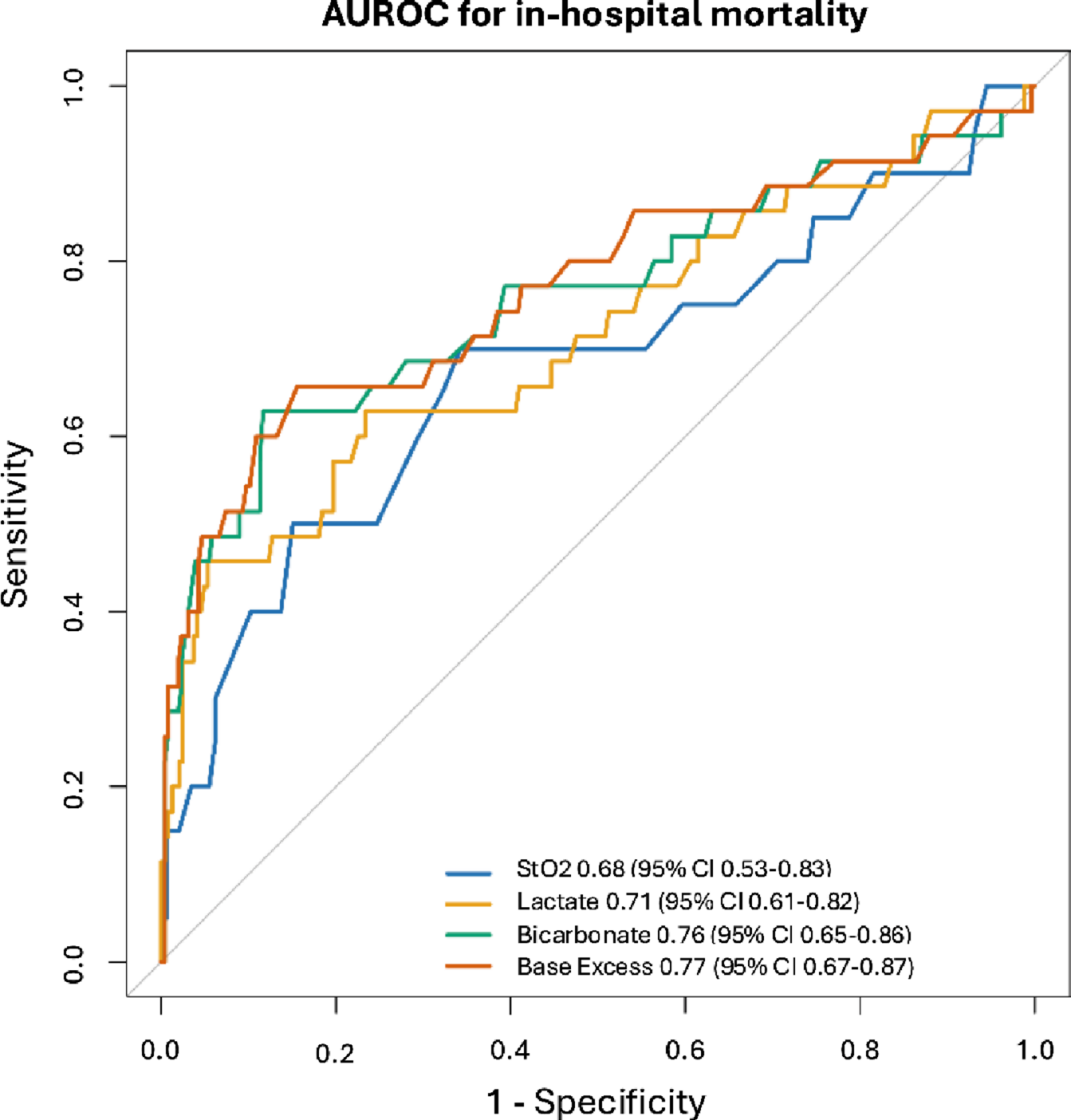
AUROC curves of StO_2_, lactate, bicarbonate, and base excess for the prediction of in-hospital mortality. *AUROC* area under the receiver operating curve, *StO*_2_ tissue oxygenation saturation, *CI* confidence interval.

The AUROC curves for in-hospital mortality for HSI StO₂, lactate, bicarbonate, and base excess overlapped (Fig. 4). Pairwise comparisons using DeLong’s test revealed no statistically significant differences among the parameters (Table 5).

**Table 5:** Pairwise comparison of AUCs for mortality.

Parameter	AUC (95% CI)	*p*-value (vs. StO_2_)	*p*-value (vs. Lactate)	*p*-value (vs. Bicarbonate)	*p*-value (vs. BE)
HSI StO_2_	0.68 (0.53–0.83)	–	0.221	0.433	0.423
Lactate	0.71 (0.61–0.82)	0.221	–	0.328	0.243
Bicarbonate	0.76 (0.65–0.86)	0.433	0.328	–	0.145
BE	0.77 (0.67–0.87)	0.423	0.243	0.145	*–*

*AUC* area under the curve, *CI* confidence interval, *StO2* tissue oxygenation saturation, *BE* Base excess. p-values from DeLong’s test for pairwise comparison of AUCs.

### Clinical utility

After bootstrap correction, the StO₂ model showed a small positive point estimate for incremental net benefit at mortality-risk thresholds between 10.0% and 15.5%; however, the 95% confidence intervals included zero throughout this range. At a 10% threshold for retaining full trauma-team resources, the optimism-corrected incremental net benefit was 0.0021 compared with retaining resources for all patients, equivalent to approximately 1.9 fewer unnecessary full-team continuations per 100 patients (95% CI − 41.4 to 29.0) (Supplemental Fig. 4). These findings do not provide evidence of a reliable clinical benefit and should be considered exploratory.

## Discussion

This study, to our knowledge, is the first to prospectively assess the diagnostic accuracy of bedside hyperspectral imaging in a trauma resuscitation room and included 163 patients. We demonstrate no significant difference in StO₂ values between trauma patients with or without hemodynamic instability during the initial resuscitation phase in trauma management. Given that StO_2_ was significantly lower in patients with lactate > 2 mmol/l, this reinforces the notion that HSI captures physiologically meaningful perfusion deficits.

At our Level I trauma center, each resuscitation room activation triggers a full trauma team activation. A validated downgrading strategy to a reduced trauma team could help balance patient safety and efficient resource utilization^22^. Current benchmarks recommend undertriage below 5% and overtriage between 25 and 30%^23^. Currently, the RISC II score is the most accurate tool for European trauma centers with an AUC of 0.953 (95% CI 0.949–0.957) for mortality^24^. As this score incorporates laboratory parameters and elements of the AIS, it is not suitable for immediate trauma team downgrading decisions. In contrast, HSI-derived StO₂ demonstrates only moderate diagnostic accuracy, with an AUC of 0.677 (95% CI 0.529–0.825). Nevertheless, an StO₂ value ≤ 54% had an NP of 94% for in-hospital mortality, suggesting a clinically relevant cutoff. This finding indicates that HSI can be used as an adjunct to clinical judgment for early downgrading. The high NPV did not translate into a robust net clinical benefit after internal validation. Given the influence of the prevalence on the NPV, these findings need to be validated in other settings.

Furthermore, we found that the AUC of HSI-derived StO₂ was comparable to that of well-established blood gas parameters for predicting in-hospital mortality. These findings support the potential role of objective, observer-independent, non-invasive assessment of microcirculation, especially tissue oxygenation, using hyperspectral imaging as a valuable adjunct to conventional monitoring in the early evaluation of trauma patients. Importantly, it can be measured rapidly during a single non-invasive assessment, potentially providing a practical tool to support trauma team downgrading or triage decisions. Any team member can perform HSI measurement without a relevant delay in the primary treatment^18^.

An association between injury severity and circulating catecholamine levels has been reported across multiple studies^25–28^. Our prior pilot study showed that even minor trauma was associated with lower StO₂ values compared with healthy controls, likely due to stress-induced endogenous catecholamine release, which causes peripheral vasoconstriction and reduced microcirculatory perfusion^18^. This effect is likely also relevant in the present study and may have contributed to the lack of significant differences in tissue oxygenation saturation between the hemodynamic instability and stability groups. This trauma-related adrenergic response may therefore have reduced the contrast between groups at the first measurement, whereas measurements at timepoint three (after 24 h) may better reflect persistent or progressive microcirculatory impairment after the initial stress response and early resuscitative interventions. So far, HSI parameters of the palm and fingertips have been investigated in 26 healthy individuals by Katzenschlager et al. and in an additional 25 healthy individuals by Dietrich et al.^17,18^. However, standardized reference values for HSI-derived parameters in healthy humans are lacking. In contrast, normal ranges have been reported in a porcine model, not only for skin but across 20 different organs^29^. This knowledge gap needs to be further addressed in future research to enable better discrimination between healthy and injured individuals and potentially to support a graded assessment approach. Such efforts may also help identify the most suitable anatomical site for HSI measurements. Also, in contrast to the pilot study, ambient lighting conditions were intentionally left unchanged to further enhance practicality and increase acceptance among the trauma team, but this might have slightly influenced the measurements.

Dietrich et al. demonstrated that HSI reliably detects hemorrhagic shock in a porcine model and dynamically captures microcirculatory changes during fluid and catecholamine resuscitation^15^. The key findings of Dietrich et al. demonstrated that StO₂ and NIR decrease significantly during hemorrhagic shock and increase to approximately preshock levels following fluid resuscitation. Catecholamine administration resulted in a dose-dependent aggravation of shock-induced reduction of StO₂^15^. In our hemodynamic instability cohort, we likewise observed reduced StO₂ and NIR values, unchanged THI, and slightly higher TWI. Although these differences did not reach statistical significance, the overall trend aligns with the findings of Dietrich et al. In accordance with this, it is reasonable to assume that a subgroup analysis of patients with hemorrhagic shock in our study would have revealed more pronounced differences in StO₂. However, due to the small and heterogeneous hemodynamic instability group, a subgroup analysis was not feasible. Moreover, prehospital interventions, particularly transfusion of blood components, may have influenced microcirculation before arrival, further limiting the ability to discriminate between groups in StO₂ at the first measurement. Aggressive fluid resuscitation of hemodynamically unstable patients may have promoted tissue edema, thereby impairing oxygen diffusion and tissue oxygenation, potentially in combination with the vasoconstrictive effects of prolonged catecholamine therapy, accounting for our observations on the third HSI measurements. In addition, glycocalyx shedding, although not specifically investigated in this study, may also have contributed to capillary leak, tissue edema, and impaired microcirculation during the later course after trauma^5^.

In sepsis, similar alterations in HSI parameters have been reported, characterized by a significant reduction in StO₂ accompanied by increased THI and TWI values^17,30,30^. The rise in THI may reflect venous congestion^15,17^ and has been identified as a strong predictor of 28-day mortality in septic patients^17^.

Several limitations should be acknowledged. (A) The single-center design of our study limits the external validity of the findings. (B) Slow patient recruitment led to early study termination, resulting in an underpowered hemodynamic instability group and reducing our ability to detect significant differences. Contributing factors included the lower-than-expected ratio of hemodynamically stable to unstable patients (approximately 20:1 rather than the assumed 5:1 in the sample size calculation) and a substantially higher number of patients without informed consent due to trauma center transfers. (C) To ensure comparability of HSI parameters, we retrospectively excluded severely injured patients with tourniquets measured at the alternative medial thigh site due to observed site-related effects.

Most of these excluded patients were in hemorrhagic shock and would have made a meaningful contribution to the hemodynamic instability cohort. (D) The analysis of the primary endpoint was based on group allocation at arrival in the resuscitation room. However, as patients’ hemodynamic status is dynamic and influenced by multiple factors, this approach may have introduced allocation bias. Contributing factors include heterogeneous trauma mechanisms and injury severity among patients, individual variations in compensatory mechanisms, and differences in the timing of hemodynamic deterioration upon arrival. As a result, some patients initially classified in the hemodynamic stability group were severely injured with a high ISS and met hemodynamic instability criteria shortly after group allocation. This overlap likely reduced the discriminative power of StO_2_ between both groups. Future studies could adopt a multicenter design to increase the number of hemodynamically unstable patients and improve statistical power, as well as assess near-infrared spectroscopy-based vascular occlusion tests (VOT), using a modified HSI protocol to compute HSI-derived VOT across all four tissue indices.

## Conclusion

In this observational study, due to the limited sample size, the absence of statistical significance between the two groups for StO_2_ should be interpreted as inconclusive rather than evidence of no effect. Nevertheless, StO₂ was significantly lower in patients with lactate levels > 2 mmol/L, indicating that HSI can detect clinically relevant perfusion deficits. Importantly, HSI provides a high NPV for in-hospital mortality, supporting its potential as a real-time decision-making tool for trauma team downgrading.

## Supplementary Information

Below is the link to the electronic supplementary material.

Supplementary Material 1

## Data Availability

The anonymized data set is available from the corresponding authors upon reasonable request.

## Abbreviations

AIS: Abbreviated Injury Scale
AUC: Area under the curve
AUROC: Area under the receiver operating characteristic
BE: Base excess
CRT: Capillary refill time
CI: Confidence intervals
EMS: Emergency Medical Service
GCS: Glasgow Coma Scale
Hb: Hemoglobin
HIS: Hyperspectral imaging
ISS: Injury Severity Score
ICU: Intensive care unit
IQR: Interquartile Range
NIR: Near infrared perfusion index
NPV: Negative predictive values
OR: Operating room
SpO_2_: Peripheral saturation
PPV: Positive predictive values
REBOA: Resuscitative Endovascular Balloon Occlusion of the Aorta
ROC: Receiver-operating characteristic
RGB: Red-Green-Blue
ROIs: Regions of interest
RISC II: Revised Injury Severity Classification II
SD: Standard deviations
STROBE: Strengthening the Reporting of Observational Studies in Epidemiology
THI: Tissue hemoglobin index
StO_2_: Tissue oxygenation saturation
TWI: Tissue water index
TBI: Traumatic brain injury
VOT: Vascular occlusion test
