# Supplementary material for: Hyperspectral imaging for objective microcirculatory assessment in trauma patients
Source: Sci Rep. 2026 Aug 6;16:24338. doi: 10.1038/s41598-026-66067-2 (PMC13448706; doi:10.1038/s41598-026-66067-2)
Supplement: Supplementary file 1 [file 41598_2026_66067_MOESM1_ESM.docx]

Supplementary Material to

Hyperspectral imaging for objective microcirculatory assessment in trauma patients

Marita Klein^1^, Maik von der Forst^1^, Nikolai Kaltschmidt^1^, Frank Weilbacher^1^, Louis de Re^1^, Christine Leowardi^2,3^, Hans Thomas Hölzer^1^, Tim Niklas Bewersdorf^4^, Maximilian Dietrich^1^, Felix C.F. Schmitt^1^, Markus A. Weigand^1^, Erik Popp^1^, Stephan Katzenschlager^1,3,#^

1 Heidelberg University, Medical Faculty Heidelberg, Department of Anesthesiology, Heidelberg, Germany

2 Heidelberg University, Medical Faculty Heidelberg, Department of General, Visceral, and Transplantation Surgery, Heidelberg, Germany

3 Heidelberg University, Medical Faculty Heidelberg, Division of Emergency Medicine, Heidelberg, Germany

4 Heidelberg University, Medical Faculty Heidelberg, Heidelberg Trauma Research Group, Clinic for Trauma and Reconstructive Surgery, Center for Orthopedics, Trauma Surgery and Spinal cord Injury, Heidelberg, Germany

\# correspondence to

Dr. med. Stephan Katzenschlager

Department of Anesthesiology, Heidelberg University Hospital, Germany

Im Neuenheimer Feld 420, 69120 Heidelberg, Germany

E-mail: [stephan.katzenschlager@med.uni-heidelberg.de](mailto:maximilian.dietrich@med.uni-heidelberg.de)

Phone: +49 6221 56 39683

Table of Contents

[Supplement Checklist 1	1](#_Toc236131802)

[Supplemental Figure 1 - Experimental setup using the Tivita® Tissue System Hyperspectral Imaging camera (Diaspective Vision GmbH, Am Salzhaff, Germany)	1](#_Toc236131803)

[Supplemental Figure 2 - Tivita® Tissue System Hyperspectral Imaging camera (Diaspective Vision GmbH, Am Salzhaff, Germany)	2](#_Toc236131804)

[Supplemental Figure 3 – Representative Hyperspectral Image	3](#_Toc236131805)

[Supplemental Figure 4 – Decision-curve analysis of initial tissue oxygenation saturation for predicting in-hospital mortality	4](#_Toc236131806)

[Supplemental Table 1 – Applied criteria for trauma team activation by the German S3 guideline on polytrauma management	5](#_Toc236131807)

[Supplemental Table 2 – Collected data points	6](#_Toc236131808)

[Supplemental Table 3 – Second HSI Measurement	7](#_Toc236131809)

[Supplemental Table 4 – Third HSI Measurement	7](#_Toc236131810)

[Supplemental Table 5 – Comparison of HSI values according to shock index	8](#_Toc236131811)

[Supplemental Table 6 – StO_2_ threshold calculation	8](#_Toc236131812)

[References	8](#_Toc236131813)

# Supplement Checklist 1

STROBE Statement—Checklist of items that should be included in reports of ***cohort studies***

|  | Item No | Recommendation | Section, Page Number |
| --- | --- | --- | --- |
| **Title and abstract** | 1 | (*a*) Indicate the study’s design with a commonly used term in the title or the abstract | Title. #1 |
|  |  | (*b*) Provide in the abstract an informative and balanced summary of what was done and what was found | Abstract, #2 |
| Introduction | | |  |
| Background/rationale | 2 | Explain the scientific background and rationale for the investigation being reported | Introduction, #3 |
| Objectives | 3 | State specific objectives, including any prespecified hypotheses | Introduction, #4 |
| Methods | | |  |
| Study design | 4 | Present key elements of study design early in the paper | Methods, #4 |
| Setting | 5 | Describe the setting, locations, and relevant dates, including periods of recruitment, exposure, follow-up, and data collection | Methods, #4 |
| Participants | 6 | (*a*) Give the eligibility criteria, and the sources and methods of selection of participants. Describe methods of follow-up | Methods/Inclusion criteria, #4-6 |
|  |  | (*b*) For matched studies, give matching criteria and number of exposed and unexposed | N/A |
| Variables | 7 | Clearly define all outcomes, exposures, predictors, potential confounders, and effect modifiers. Give diagnostic criteria, if applicable | Methods, #6-10 |
| Data sources/ measurement | 8* | For each variable of interest, give sources of data and details of methods of assessment (measurement). Describe comparability of assessment methods if there is more than one group | *Methods, #9-10* |
| Bias | 9 | Describe any efforts to address potential sources of bias | Methods/HSI measurements, #7-8 |
| Study size | 10 | Explain how the study size was arrived at | Methods/Sample size, #8 |
| Quantitative variables | 11 | Explain how quantitative variables were handled in the analyses. If applicable, describe which groupings were chosen and why | Methods/statistical analysis, #9 |
| Statistical methods | 12 | (*a*) Describe all statistical methods, including those used to control for confounding | Methods/statistical analysis, #9-10 |
|  |  | (*b*) Describe any methods used to examine subgroups and interactions | Methods/statistical analysis, #9-10 |
|  |  | (*c*) Explain how missing data were addressed | Methods/statistical analysis, #9-10 |
|  |  | (*d*) If applicable, explain how loss to follow-up was addressed | Methods/statistical analysis, #9-10 |
|  |  | (*e*) Describe any sensitivity analyses | Methods/statistical analysis, #9-10 |
| Results | | |  |
| Participants | 13* | (a) Report numbers of individuals at each stage of study—eg numbers potentially eligible, examined for eligibility, confirmed eligible, included in the study, completing follow-up, and analysed | Results/Demographics, #10 |
|  |  | (b) Give reasons for non-participation at each stage | Results/Demographics, #10 |
|  |  | (c) Consider use of a flow diagram | Figure 1 |
| Descriptive data | 14* | (a) Give characteristics of study participants (eg demographic, clinical, social) and information on exposures and potential confounders | Results/Demographics, #11-12, Table 1 |
|  |  | (b) Indicate number of participants with missing data for each variable of interest | Table 2 |
|  |  | (c) Summarise follow-up time (eg, average and total amount) | Table 2 |
| Outcome data | 15* | Report numbers of outcome events or summary measures over time | Results/Hyperspectral imaging, #14 |
| Main results | 16 | (*a*) Give unadjusted estimates and, if applicable, confounder-adjusted estimates and their precision (eg, 95% confidence interval). Make clear which confounders were adjusted for and why they were included | Results/Hyperspectral imaging, #14, Table 3, Figure 2 |
|  |  | (*b*) Report category boundaries when continuous variables were categorized | Figure 2 |
|  |  | (*c*) If relevant, consider translating estimates of relative risk into absolute risk for a meaningful time period | N/A |
| Other analyses | 17 | Report other analyses done—eg analyses of subgroups and interactions, and sensitivity analyses | Table 4, Figure 3, Figure 4, Table 5, Supplement Table 5-6 |
| Discussion | | |  |
| Key results | 18 | Summarise key results with reference to study objectives | Discussion, #17-18 |
| Limitations | 19 | Discuss limitations of the study, taking into account sources of potential bias or imprecision. Discuss both direction and magnitude of any potential bias | Discussion #20 |
| Interpretation | 20 | Give a cautious overall interpretation of results considering objectives, limitations, multiplicity of analyses, results from similar studies, and other relevant evidence | Discussion #17-18 |
| Generalisability | 21 | Discuss the generalisability (external validity) of the study results | Discussion #19-20 |
| Other information | | |  |
| Funding | 22 | Give the source of funding and the role of the funders for the present study and, if applicable, for the original study on which the present article is based | Declarations/Funding, #22 |

*Give information separately for exposed and unexposed groups.

## Supplemental Figure 1 - Experimental setup using the Tivita® Tissue System Hyperspectral Imaging camera (Diaspective Vision GmbH, Am Salzhaff, Germany)

## Supplemental Figure 2 - Tivita® Tissue System Hyperspectral Imaging camera (Diaspective Vision GmbH, Am Salzhaff, Germany)

## Supplemental Figure 3 – Representative Hyperspectral Image

*A circular ROI of 45 units was used for quantitative analysis, and additionally the color-coded scale can be used for qualitative analysis with red/yellow tones indicating higher values (50–100) and green/blue tones representing lower values (0–50). Abbreviations: RGB = Red-Green-Blue image StO_2_ = tissue oxygenation saturation, THI = tissue hemoglobin index, NIR = near infrared perfusion index, TWI = tissue water index, ROI = region of interest.*

## Supplemental Figure 4 – Decision-curve analysis of initial tissue oxygenation saturation for predicting in-hospital mortality

The solid blue line represents the bootstrap optimism-corrected net benefit of the StO₂ model, the dashed blue line represents apparent net benefit, and the shaded area represents the pointwise 95% confidence interval based on 1,000 bootstrap repetitions. The orange and black lines represent strategies of retaining full trauma-team resources for all and no patients, respectively.

## Supplemental Table 1 – Applied criteria for trauma team activation by the German S3 guideline on polytrauma management

| Criteria with highest level of recommendation | Elements |
| --- | --- |
| Pathological (vital) signs | SpO_2_ < 90%, need for airway management, systolic blood pressure < 90 mmHg, heart rate > 120/min, shock index > 0.9, positive eFAST screening, GCS ≤ 12, hypothermia < 35 °C |
| Specific injury patterns  Need for critical prehospital interventions | Unstable thorax or pelvis, penetrating torso/neck trauma, proximal amputations, spinal injuries with signs of neurological deficits  Airway management, chest decompression, catecholamine administration, pericardiocentesis, use of tourniquet |
| **Criteria with high level of recommendation** |  |
| Special injuries | ≥ 2 proximal major long-bone fractures, burns > 20 % total body surface area grade ≥ 2b |
| Mechanism | Fall > 3 m, motor vehicle collision with ejection or long-bone fracture |
| Age | Liberal approach for geriatric patients with: systolic blood pressure < 100 mmHg, (known or suspected) TBI and GCS ≤ 14, ≥ 2 injured body regions, ≥ 1 long-bone fracture after motor vehicle collision |

Abbreviations: SpO_2_ = peripheral saturation, eFAST = extended Focused Assessment with Sonography for Trauma, GCS = Glasgow Coma Scale, TBI = traumatic brain injury. Note: Fulfillment of a single criterion is sufficient to justify trauma team activation [1].

## Supplemental Table 2 – Collected data points

| Category of collected Data points | Elements |
| --- | --- |
| Patient history | Age, sex, body weight, height, body surface area, pre-existing conditions, long-term medication, allergies, alcohol/nicotine/drugs, previous operations |
|  |  |
| Current incident | Date of screening, start of symptoms, status of enrolment, mechanism of trauma, injuries |
|  |  |
| Vital signs on admission (1^st^ HSI measurement), after resuscitation room phase (2^nd^ HSI measurement), 24 hrs (3^rd^ HSI measurement) | Heart rate, respiratory rate, systolic blood pressure, diastolic blood pressure, mean arterial blood pressure, body temperature, skin temperature palm, SpO_2_, blood glucose, ECG rhythm, GCS, CRT, ISS |
|  |  |
| Active medication on all three measurement time points | Time of arrival, time of HSI measurements, noradrenaline, adrenaline, vasopressin, dobutamine, other catecholamines, sufentanil, fentanyl, midazolam, esketamine, propofol, rocuronium, piritramide, other sedatives and analgetics, other medication, crystalloids, colloids, red blood cell transfusion, plasma transfusion, prothrombin complex, fibrinogen |
|  |  |
| Imaging | eFAST, X-ray, CT-Scan |
|  |  |
| Treatment and interventions (prehospital and ER) | Needle decompression, pelvic sling, tourniquet, thoracostomy, REBOA, clamshell, pericardiocentesis, intubation, other |
|  |  |
| HSI measurements | Time, Fitzpatrick Score, ROI, StO_2_, NIR, THI, TWI |
|  |  |
| Blood gas analyses | pH, pCO_2_, pO_2_, bicarbonate, base excess, hematocrit, hemoglobin, Na^+^, K^+^, Ca^++^, Cl^-^, blood glucose, lactate, arterial/venous/capillary, time |
|  |  |
| Laboratory blood parameters | Na^+^, K^+^, Ca^++^, Cl^-^, creatinine, GFR (CKD-EPI), urea, blood glucose, CK, troponin T, CRP, hemolysis, lipemia, icteric, leukocytes, erythrocytes, hemoglobin, hematocrit, MCV, MCH, MCHC, RCDW, thrombocytes, Quick, INR, pTT, D-dimers, thrombin time, fibrinogen |
|  |  |
| Immediate Outcome | ICU admission, ICU duration, ventilation days, non-ICU observation, OR, death, same day discharge, diagnoses |
|  |  |
| 30-day follow-up | Survival status, CPC, mRScale, GOS |

*Abbreviations: HSI = Hyperspectral Imaging, SpO2 = peripheral saturation, ECG = electrocardiogram, GCS = Glasgow Coma Scale, CRT = capillary refill time, ISS = Injury Severity Score, eFAST = extended Focused Assessment with sonography for trauma, CT-scan = computed tomography scan, REBOA = Resuscitative Endovascular Balloon Occlusion of the Aorta, ROI = region of interest, StO_2_ = tissue oxygenation saturation, NIR = near infrared perfusion index, THI = tissue hemoglobin index, THI = tissue water index, pCO_2_ = partial pressure of carbon dioxide, pO_2_ = partial pressure of oxygen , GFR CKD-EPI= glomerular filtration rate Chronic Kidney Disease Epidemiology Collaboration, CK = creatine kinase, CRP = C-reactive protein, MCV = mean corpuscular volume, MCH = mean corpuscular hemoglobin, MCHC = mean corpuscular hemoglobin concentration, RCDW = red cell distribution width, INR = international normalized ratio, partial thromboplastin time, ICU = intensive care unit, OR = operating room, CPC = cerebral performance category, mRScale = modified Rankin Scale, GOS = Glasgow Outcome Scale.*

|  | Hemodynamic instability (n = 23) | Hemodynamic stability (n = 137) | Difference (95% CI) | p-value |
| --- | --- | --- | --- | --- |
| StO₂ [%], mean (SD) | 58.04 (14.17) | 57.42 (9.69) | -0.62 (-5.26 to 4.02) | 0.792 |
| THI [U], mean (SD) NIR [U], mean (SD) TWI [U], mean (SD) | 20.39 (13.60)  49.74 (9.73) 62.13 (8.55) | 19.50 (11.58)  51.43 (8.73) 58.23 (10.04) | -0.89 (-6.18 to 4.40)  1.69 (-2.26 to 5.64) -3.90 (-9.96 to 0.48) | 0.741  0.399 0.080 |

## Supplemental Table 3 – Second HSI Measurement

*Abbreviations: HSI = Hyperspectral Imaging, StO_2_ = tissue oxygenation saturation, THI = tissue hemoglobin index, NIR = near infrared perfusion index, TWI = tissue water index, SD = standard deviation, CI = Confidence Interval.*

## Supplemental Table 4 – Third HSI Measurement

|  | Hemodynamic instability (n = 10) | Hemodynamic stability (n = 34) | Difference (95% CI) | p-value |
| --- | --- | --- | --- | --- |
| StO₂ [%], mean (SD) | 52.60 (8.83) | 62.68 (11.46) | 10.08 (2.13 to 18.03) | 0.014 |
| THI [U], mean (SD) NIR [U], mean (SD) TWI [U], mean (SD) | 11.50 (8.05)  53.40 (10.32) 67.00 (6.75) | 15.23 (9.58)  58.15 (6.90) 63.88 (10.03) | 3.80 (-2.94 to 10.52)  4.75 (-0.89 to 10.38) -3.12 (-8.29 to 3.72) | 0.262  0.096 0.363 |

*Abbreviations: HSI = Hyperspectral Imaging, StO_2_ = tissue oxygenation saturation, THI = tissue hemoglobin index, NIR = near infrared perfusion index, TWI = tissue water index, SD = standard deviation, CI = Confidence Interval.*

## Supplemental Table 5 – Comparison of HSI values according to shock index

|  | Shock Index ≥0.9 (n = 31) | Shock Index <0.9 (n = 132) | Difference (95% CI) | p-value |
| --- | --- | --- | --- | --- |
| StO₂ [%], mean (SD) | 56.5 (13.0) | 59.8 (11.4) | -3.25 (-8.36 to 1.86) | 0.206 |
| THI [U], mean (SD) NIR [U], mean (SD) TWI [U], mean (SD) | 22.0 (14.7)  53.4 (11.5) 61.0 (8.9) | 20.5 (11.7)  54.5 (9.4) 61.9 (7.6) | 1.55 (-4.16 to. 7.25)  -1.11 (-5.60 to 3.39) -0.93 (-2.24 to 4.09) | 0.587  0.622 0.748 |

*Abbreviations: StO_2_ = tissue oxygenation saturation, PPV = positive predictive value, NPV = negative predictive value.*

## Supplemental Table 6 – StO_2_ threshold calculation

| Cutoff StO_2_ | Sensitivity | Specificity | PPV | NPV | LR + (95% CI) | LR – (95% CI) | Youden Index |
| --- | --- | --- | --- | --- | --- | --- | --- |
| 54 | 0.70 | 0.66 | 0.22 | 0.94 | 1.92 (1.26-2.91) | 0.53 (0.28-1.03) | 0.36 |
| 48  53  49  52 | 0.50  0.65  0.50 0.60 | 0.85  0.68  0.82 0.71 | 0.31  0.22  0.28 0.22 | 0.93  0.93  0.92 0.93 | 2.84 (1.42-5.68)  1.85 (1.16-2.94)  2.38 (1.22-4.67)  1.82 (1.09-3.06) | 0.68 (0.46-1.03)  0.60 (0.33-1.07)  0.71 (0.47-1.06)  0.66 (0.39-1.10) | 0.35  0.33  0.32 0.31 |

*Abbreviations: StO_2_ = tissue oxygenation saturation, PPV = positive predictive value, NPV = negative predictive value, LR + = positive likelihood ratio, LR - = negative likelihood ratio, CI = confidence interval.*

References

1.	Deutsche Gesellschaft für Unfallchirurgie e.V. (DGU), Deutsche Gesellschaft für Orthopädie & Unfallchirurgie e.V. (DGOU). S3-Leitlinie Polytrauma/Schwerverletzten-Behandlung: Deutsche Gesellschaft für Unfallchirurgie e.V. https://www.awmf.org/leitlinien/detail/ll/187-023.html.
